# Association of d-dimer levels with in-hospital death and multi-vessel coronary artery disease in patients with non–ST-segment elevation acute coronary syndrome

**DOI:** 10.3389/fmed.2025.1680631

**Published:** 2025-11-19

**Authors:** Ning Zhang, Baorong Niu

**Affiliations:** 1 Department of Cardiology, Beijing Anzhen Hospital, Capital Medical University, Beijing, China; 2 Department of Echocardiography, Beijing Anzhen Hospital, Capital Medical University, Beijing, China

**Keywords:** non–ST-segment elevation acute coronary syndrome, d-dimer dimerization, risk factors, in-hospital death, extent of coronary artery lesions

## Abstract

**Introduction:**

To determine the association of d-dimer levels with in-hospital death and coronary artery disease (CAD) in patients with non–ST-segment elevation acute coronary syndrome (NSTEACS).

**Methods:**

We retrospectively analyzed the data of 803 NSTEACS patients admitted to Beijing Anzhen Hospital between January 2022 and January 2024. Demographic, clinical, and laboratory data were compared between survivors (792 patients) and non-survivors (11 patients). Risk factors for in-hospital death were analyzed. The cutoff plasma d-dimer level was determined using the Youden index to predict in-hospital death in patients with NSTEACS, and the rates of in-hospital mortality and multi-vessel CAD (2-vessel + 3-vessel disease) were compared between patients with high (103 patients) and low d-dimer levels (700 patients).

**Results:**

D-dimer [odds ratio (OR): 1.003, *p* = 0.033, 95% confidence interval (CI): 1.000–1.006], Global Registry of Acute Coronary Events score (OR: 3.174, *p* < 0.001, 95% CI: 1.686–5.977), multi-vessel CAD (OR: 6.118, *p* = 0.013, 95% CI: 1.468–25.499), and Thrombolysis in Myocardial Infarction score (OR: 1.060, *p* = 0.002, 95% CI: 1.022–1.099) were independently associated with in-hospital death. The area under the receiver operating characteristic curve of d-dimer was 0.781 (*p* = 0.001, 95% CI: 0.634–0.927). The maximum Youden index was 0.607, with a cutoff of 504 ng/mL distinguishing high and low risk of in-hospital death in NSTEACS patients. In-hospital mortality (8/103 *vs*. 3/700, *p* < 0.001) and multi-vessel CAD rates (47/103 *vs*. 222/700, *p* = 0.003) were higher in the high d-dimer group.

**Conclusion:**

The higher the d-dimer level in NSTEACS patients, the higher the risk of in-hospital death and the higher the probability of multi-vessel CAD. D-dimer levels were significantly associated with in-hospital death and multi-vessel CAD in NSTEACS patients.

## Introduction

Acute coronary syndrome (ACS) is characterized by acute myocardial ischemia due to the rupture and erosion of atherosclerotic plaques in the coronary arteries, followed by thrombus formation. ACS is a severe form of coronary heart disease that is associated with major adverse cardiac events, including short-term in-hospital mortality and long-term mortality. Non–ST-segment elevation acute coronary syndrome (NSTEACS) is a type of ACS that includes non–ST-segment elevation myocardial infarction (NSTEMI) and unstable angina pectoris (UAP). NSTEACS accounts for approximately two-thirds of all hospital admissions for ACS in the United States each year (1), and is characterized by high incidence and mortality rates. The clinical presentation and prognosis of NSTEACS vary widely. Current risk-stratification systems for assessing the prognosis of NSTEACS patients exhibit inconsistent discriminative abilities, particularly in predicting the angiographic severity of coronary artery disease (CAD) (2–5). Therefore, new evaluation methods are needed to improve the predictive capabilities of risk-stratification methods for NSTEACS.

D-dimer is a biomarker reflecting the coagulation state and thrombus formation (6). Elevated d-dimer levels have been associated with vulnerable plaques (7) and no reflow after coronary intervention (8, 9). High d-dimer levels have also been correlated with increased long-term mortality in patients with stable coronary heart disease (10, 11) and ST-segment elevation myocardial infarction (STEMI) (12, 13). However, few studies have investigated the relationship between d-dimer levels and NSTEACS, particularly in the context of predicting the extent of coronary artery lesions. NSTEACS includes both NSTEMI and UAP. Unlike troponin, d-dimer, as a marker of fibrinolysis, has a unique advantage in reflecting thrombus formation. This is especially true in patients with UAP, where myocardial necrosis is not prominent and troponin levels typically do not significantly rise. D-dimer can provide additional information regarding thrombus burden and the risk of adverse cardiovascular events. Therefore, D-dimer plays a role in NSTEACS not only by complementing the limitations of troponin testing but also by offering greater predictive value in UAP patients. The number of affected coronary vessels (1-vessel, 2-vessel, and 3-vessel disease) provides a practical and straightforward approach for evaluating the extent of CAD, and aligns well with everyday clinical decision-making (for example, for percutaneous coronary intervention and coronary artery bypass grafting). Therefore, this study aimed to determine whether d-dimer levels are associated with in-hospital death and multi-vessel CAD in NSTEACS patients. We hope that our findings will facilitate the selection of more appropriate treatment strategies for NSTEACS and improve patient prognosis.

## Materials and methods

### Study design and subjects

This retrospective observational study enrolled NSTEACS patients who were admitted to Anzhen Hospital between January 2022 and January 2024. The inclusion criteria were as follows: (1) patients aged ≥ 18 years who met the 2020 European Society of Cardiology diagnostic criteria for NSTEACS (14), (2) patients who received standard medical therapy according to current practice guidelines (consisting of aspirin, clopidogrel/ticagrelor, statins, *β*-blockers, and/or angiotensin convertase enzyme inhibitors/angiotensin receptor blockers/angiotensin receptor-neprilysin inhibitor as appropriate), and (3) patients who underwent coronary angiography for the assessment of coronary status. The following exclusion criteria were applied: (1) myocarditis or cardiomyopathy, (2) complications involving the liver or the lungs, acute brain injury, or acute pancreatitis, (3) a documented history of thrombotic diseases, including deep vein thrombosis (ICD-10: I82), pulmonary embolism (ICD-10: I26), cerebral infarction due to arterial thrombosis (ICD-10: I63), myocardial infarction due to coronary thrombosis (ICD-10: I21.9), and arterial embolism and thrombosis (ICD-10: I74), and (4) pregnancy, acute infectious diseases, and malignancies. The exclusion criteria were rigorously applied through a comprehensive review of medical records, laboratory findings (coagulation profiles), imaging studies (venous ultrasonography, echocardiography), and when necessary, consultations with relevant specialists.

### Ethical considerations

This study conformed to the Declaration of Helsinki and its amendments, and was approved by the ethics committee of Beijing Anzhen Hospital, Capital Medical University (approval number: 2024012X). This retrospective study only used data contained in the patients’ medical records, so the need for obtaining informed consent was waived by the ethics committee of our hospital.

### Data collection

Demographic and clinical data of the patients were collected, including age, gender, medical history, vital signs measured upon admission, laboratory measurements (e.g., routine blood tests, cardiac biomarkers, arterial lactate levels, lipid profile, blood glucose, liver- and kidney-function tests, serum B-type natriuretic peptide [BNP], albumin, high-sensitivity C-reactive protein [hs-CRP], and d-dimer), and comprehensive echocardiographic assessment of cardiac function (the echo cardiographers were blinded to the d-dimer levels). On the day of admission, the Thrombolysis in Myocardial Infarction (TIMI) and Global Registry of Acute Coronary Events (GRACE) scores were calculated, and the Gensini score was calculated based on the results of coronary angiography.

All patients included in the study had undergone D-dimer measurements. D-dimer was measured upon hospital admission, prior to any procedures. If multiple measurements were available, the initial measurement was used for analysis. D-dimer testing was performed using the Werfen ACL TOP Series analyzer, with results reported in nanograms per milliliter d-dimer units (DDU) and a defined reference range of 0–243 ng/mL DDU. The assay demonstrated good precision, with both the intra-assay and inter-assay coefficients of variation being less than 5%. All blood samples for d-dimer measurement were obtained prior to the initiation of anticoagulation therapy.

### Study outcomes

The primary outcome of the study was in-hospital death, and the secondary outcome was the extent of CAD, which was evaluated based on the number of affected coronary vessels, and categorized as 1-vessel, 2-vessel, or 3-vessel disease. Multi-vessel CAD was defined as the presence of stenosis affecting ≥70% of the vessel diameter in at least 2 major epicardial coronary arteries or ≥50% stenosis in the left main coronary artery (15, 16). To assess the reproducibility of the angiographic interpretations, a second experienced interventional cardiologist, who was blinded to the initial assessment as well as to the patients’ d-dimer levels, independently reviewed a random sample of 100 angiograms. The inter-observer agreement was excellent, with a Cohen’s kappa coefficient of 0.85 for the diagnosis of multi-vessel CAD. We also used the Gensini score to further quantify the severity of coronary lesions. The Gensini score was independently evaluated by 2 cardiologists to ensure consistency. Potential risk factors for the in-hospital death of NSTEACS patients were analyzed, and the impact of d-dimer levels on the rates of in-hospital death and severity of CAD was evaluated.

### Statistical analysis

Data were presented as numbers (percentages), mean ± standard deviation, or median (interquartile range), and analyzed using SPSS *v*26.0 statistical software with the chi-square test, Student *t*-test, and the Mann–Whitney U test. Given that some variables had fewer than 5 data points, we applied the Fisher exact test in addition to the chi-squared test to ensure the robustness of the results. Due to the low number of in-hospital deaths, univariable logistic regression was employed to assess the independent associations between prespecified variables and the outcomes of interest, in order to avoid potential overfitting associated with conventional multivariable logistic regression. The variables were selected *a priori* based on their clinical relevance and the study objective to identify simple, readily available supplementary indicators, including d-dimer, multi-vessel CAD, GRACE score, TIMI score, and Gensini score. The potential association of each indicator with in-hospital death among NSTEACS patients was also analyzed using receiver operating characteristic (ROC) curves, and the area under the curve (AUC) was calculated. Variables that did not follow a normal distribution were log-transformed to achieve a normal distribution. The transformed variables were then subjected to correlation analysis, and scatter plots were generated. A *p*-value < 0.05 was considered statistically significant.

## Results

### Study population

**Figure 1:**
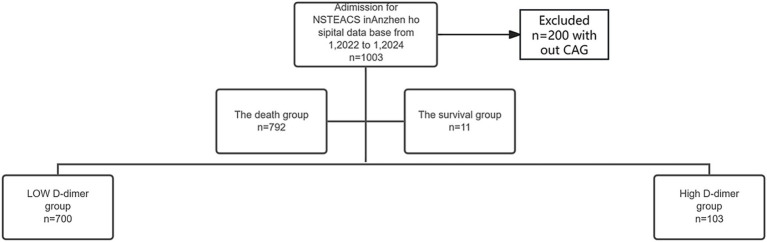
Flowchart showing the study design. NSTEACS, non–ST-segment elevation acute coronary syndrome; CAG, coronary angiography.

During the study period, 1,003 NSTEACS patients were admitted to our hospital (Figure 1). We excluded 200 patients who did not undergo coronary angiography. The remaining 803 NSTEACS patients were enrolled in this study. Their mean age was 60.00 ± 9.929 years, and 72.20% of the patients were men. In total, 11 in-hospital deaths occurred in this study, and 792 patients were discharged successfully. No significant differences were found in the baseline demographic characteristics of the survivors and non-survivors. The majority of the non-surviving patients had acute NSTEMI and multi-vessel CAD.

### Comparison of the clinical data of survivors and non-survivors

Compared to the survivors, the non-survivors had a higher prevalence of diabetes, a faster heart rate, higher arterial lactate levels, higher Glucose levels, higher myocardial marker levels, higher d-dimer levels, higher hs-CRP levels, higher BNP levels, lower ejection fraction (EF) values, higher Gensini scores, higher GRACE scores, higher TIMI scores, higher rates of acute NSTEMI, and higher rates of multi-vessel CAD (all *p* < 0.005; Table 1).

**Table 1:** Demographic and clinical characteristics and laboratory results of patients in the death and survival groups.

Parameter	Survival (*n* = 792)	Death (*n* = 11)	t/χ^2^/Z	*P*
Age (years, x ± s)	59.98 ± 9.91	61.36 ± 11.91	t = −0.460	0.646
Male sex (*n*, %)	573 (72.35)	7 (63.64)	χ^2^ = 0.411	0.522
Smoking (*n*, %)	330 (41.67)	3 (27.27)	χ^2^ = 5.470	0.065
Hypertension (*n*, %)	483 (61.00)	8 (72.73)	χ^2^ = 0.614	0.433
Diabetes (*n*, %)	235 (26.67)	7 (63.64)	χ^2^ = 5.910	0.015
Hyperlipidemia (*n*, %)	205 (25.88)	2 (18.18)	χ^2^ = 3.836	0.050
Cerebrovascular disease (*n*, %)	82 (10.35)	3 (27.27)	χ^2^ = 3.264	0.071
Family history of early-onset coronary heart disease (*n*, %)	3 (0.38)	0 (0.00)	χ^2^ = 0.042	0.838
Previous PCI (*n*, %)	174 (21.97)	1 (9.09)	χ^2^ = 1.067	0.302
Previous CABG (*n*, %)	12 (1.52)	2 (18.18)	χ^2^ = 17.489	<0.001
Pre-hospital CPR (*n*, %)	1 (0.13)	0 (0.00)	χ^2^ = 0.008	0.931
BP [mm Hg, M (P25, P75)]	133 (113, 140)	128 (117, 139)	Z = 0.151	0.880
HR [BPM, M (P25, P75)]	70 (63, 78)	80 (76, 107)	Z = −3.909	<0.001
WBC [× 10^9^/L, M (P25, P75)]	6.66 (5.56, 7.89)	6.58 (5.31, 9.11)	Z = 0.571	0.568
Hb [g/L, M (P25, P75)]	140 (129, 151)	132 (114, 153)	Z = 0.790	0.429
SCr [μmol/L, M (P25, P75)]	77.30 (66.43, 88.80)	80.00 (63.00, 103.00)	Z = −0.496	0.620
Lac [mmol/L, M (P25, P75)]	1.40 (1.10, 1.80)	1.90 (1.38, 3.00)	Z = −1.992	0.046
CK-MB [ng/mL, M (P25, P75)]	1.60 (1.00, 2.75)	3.60 (1.70, 9.10)	Z = −2.879	0.004
cTnI [ng/mL, M (P25, P75)]	0.01 (0.00, 0.41)	0.84 (0.03, 6.05)	Z = −2.688	0.007
Glucose [mmol/L, M (P25, P75)]	6.02 (5.25, 7.74)	11.35 (9.68, 14.63)	Z = −4.457	<0.001
D-dimer [ng/mL, M (P25, P75)]	62.68 (22.64, 164.05)	647.85 (44.59, 1303.04)	Z = −3.199	0.001
hs-CRP [mg/L, M (P25, P75)]	1.74 (0.63, 4.48)	16.22 (1.4, 28.58)	Z = −3.148	0.002
BNP [ng/L, M (P25, P75)]	55.00 (23.00, 131.00)	281.00 (48.53, 719.70)	Z = −2.177	0.029
EF value [%, M (P25, P75)]	63 (58, 67)	45 (40, 58)	Z = 3.717	<0.001
Type of NSTEACS (*n*, %)			χ^2^ = 15.051	<0.001
NSTEMI	274 (34.60)	10 (90.91)		
UAP	518 (65.40)	1 (9.09)		
Number of stenosed coronary vessels (*n*, %)			χ^2^ = 4.892	0.086
1-vessel disease	530 (66.92)	4 (36.36)		
2-vessel disease	128 (16.16)	4 (36.36)		
3-vessel disease	134 (16.92)	3 (27.27)		
Multi-vessel CAD (*n*, %)	262 (33.08)	7 (63.64)	χ^2^ = 4.440	0.035
Gensini score [M (P25, P75)]	30 (12.00, 57.25)	64 (48.00, 87.50)	Z = −2.710	0.030
TIMI score [M (P25, P75)]	2 (1, 3)	3 (2, 5)	Z = −4.808	<0.001
GRACE score [M (P25, P75)]	99 (85, 114)	129 (88, 180)	Z = −4.199	<0.001
lgD-dimer (x ± s)	1.83 ± 0.64	2.57 ± 0.66	t = −3.803	<0.001

Data are expressed as number (percentage), mean ± SD, or median (interquartile range), as specified. PCI, percutaneous coronary intervention; CABG, coronary artery bypass grafting; CPR, cardiopulmonary resuscitation; BP, blood pressure; HR, heart rate; BPM, beats per minute; WBC, white blood cell; Hb, hemoglobin; SCr, serum creatinine; Lac, lactate; CK-MB, creatine kinase-MB; cTnI, cardiac troponin I; hs-CRP, high-sensitivity C-reactive protein; BNP, B-type natriuretic peptide; EF, ejection fraction; NSTEACS, non–ST-segment elevation acute coronary syndrome; NSTEMI, non–ST-segment elevation myocardial infarction; UAP, unstable angina pectoris; CAD, coronary artery disease; TIMI, Thrombolysis in Myocardial Infarction; GRACE, Global Registry of Acute Coronary Events; lgD-dimer, log-transformed D-dimer values.

### Factors associated with in-hospital death among NSTEACS patients

Univariable logistic regression analysis showed that high d-dimer level (odds ratio [OR]: 1.003, *p* = 0.033, 95% confidence interval [CI]: 1.000–1.006), high TIMI score (OR: 1.060, *p* = 0.002, 95% CI: 1.022–1.099), high GRACE score (OR: 3.174, *p* < 0.001, 95% CI: 1.686–5.977), and multi-vessel CAD (OR: 6.118, *p* = 0.013, 95% CI: 1.468–25.499) were independently associated with in-hospital death (Table 2).

**Table 2:** Univariable logistic regression analysis of risk factors for in-hospital death.

Variable	β	SE	Wald value	P-value	OR	95% CI
d-Dimer	0.003	0.001	4.536	0.033	1.003	(1.000–1.006)
Multi-vessel CAD	1.811	0.728	6.186	0.013	6.118	(1.468–25.499)
GRACE score	1.155	0.323	12.800	0.000	3.174	(1.686–5.977)
TIMI score	0.058	0.019	9.770	0.002	1.060	(1.022–1.099)

SE, standard error; OR, odds ratio; 95% CI, 95% confidence interval; CAD, coronary artery disease; TIMI, Thrombolysis in Myocardial Infarction; GRACE, Global Registry of Acute Coronary Events.

**Figure 2:**
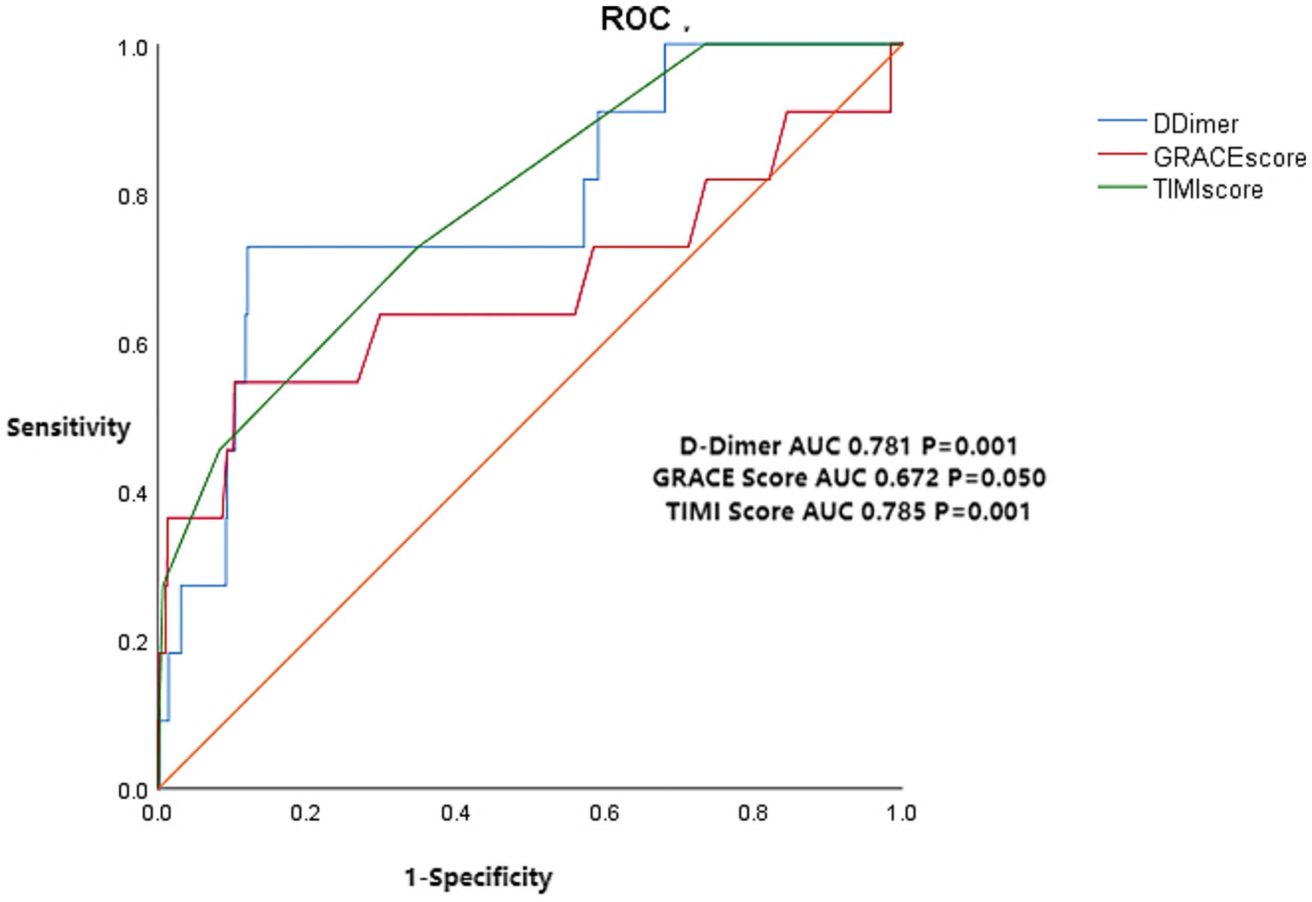
ROC curve analysis of d-dimer level, GRACE score, and TIMI score for in-hospital death in NSTEACS patients. ROC, receiver operating characteristic; AUC, area under the curve; GRACE, Global Registry of Acute Coronary Events; TIMI, Thrombolysis in Myocardial Infarction; NSTEACS, non–ST-segment elevation acute coronary syndrome.

ROC curve analyses identified the following variables as potentially valuable for predicting in-hospital death in NSTEACS patients: d-dimer level (AUC: 0.781, *p* = 0.001, 95% CI: 0.634–0.927), GRACE score (AUC: 0.672, *p* = 0.050, 95% CI: 0.459–0.885), and TIMI score (AUC: 0.785, *p* = 0.001, 95% CI: 0.649–0.920). The maximum Youden index of d-dimer to predict in-hospital death in NSTEACS patients was 0.607, with a cutoff value of 504 ng/mL (Figure 2). At this cutoff, the sensitivity and specificity of d-dimer levels were 0.727 and 0.878, respectively. Consideration was given to the trade-off between sensitivity and specificity when selecting the cutoff point. While maximizing either sensitivity or specificity could lead to a different threshold, the Youden index was used to strike a balance between these 2 metrics, ensuring an optimal overall performance of the test.

### Stratification based on d-dimer levels

Using the cutoff d-dimer value, we divided the patients into a high d-dimer group (>504 ng/mL) and a low d-dimer group (≤504 ng/mL). We utilized a d-dimer cutoff of 504 ng/mL, which is slightly above the standard threshold of 500 ng/mL, to evaluate its clinical significance in our study population. Compared to the low d-dimer group, the high d-dimer group had a higher in-hospital mortality rate, older age, faster heart rate, higher white blood cell count, higher blood creatinine levels, higher levels of myocardial markers, higher hs-CRP levels, higher BNP levels, higher Gensini scores, higher GRACE scores, higher TIMI scores, lower hemoglobin levels, lower EF values, higher rates of acute NSTEMI, and higher rates of multi-vessel CAD (all *p* < 0.005; Table 3).

**Table 3:** Demographic and clinical characteristics and laboratory results of patients in different d-dimer groups.

Parameter	D-dimer ≤ 504 ng/mL (*n* = 700)	D-dimer > 504 ng/mL (*n* = 103)	t/χ^2^/Z	*P*
In-hospital death (*n*, %)	3 (0.43)	8 (7.77)		<0.001
Age (years, x ± s)	59.68 ± 9.59	62.14 ± 11.81	t = −2.350	0.019
Male sex (*n*, %)	508 (72.57)	72 (69.90)	χ^2^ = 0.319	0.572
Smoking (*n*, %)	292 (41.71)	39 (37.86)	χ^2^ = 5.470	0.065
Hypertension (*n*, %)	421 (60.14)	70 (67.96)	χ^2^ = 2.212	0.137
Diabetes (*n*, %)	205 (29.29)	37 (25.92)	χ^2^ = 1.828	0.176
Hyperlipidemia (*n*, %)	187 (26.71)	22 (21.36)	χ^2^ = 3.836	0.050
Cerebrovascular disease (*n*, %)	73 (10.43)	12 (11.65)	χ^2^ = 0.080	0.777
Family history of early-onset coronary heart disease (*n*, %)	2 (0.29)	1 (0.97)		0.339
Previous PCI (*n*, %)	155 (22.14)	20 (19.42)	χ^2^ = 0.656	0.418
Previous CABG (*n*, %)	12 (1.71)	2 (1.94)		0.699
Pre-hospital CPR (*n*, %)	1 (0.14)	0 (0.00)		0.918
BP (mm Hg, x ± s)	128.81 ± 17.84	129.39 ± 21.16	t = −0.300	0.765
HR (BPM, x ± s)	69.99 ± 11.38	78.85 ± 17.76	t = −6.785	<0.001
WBC (×10^9^/L, x ± s)	6.75 ± 1.77	8.51 ± 3.13	t = −8.341	<0.001
Hb (g/L, x ± s)	140.39 ± 17.90	129.47 ± 20.71	t = 5.663	<0.001
SCr (μmol/L, x ± s)	82.59 ± 58.06	95.68 ± 48.08	t = −2.181	0.029
Lac (mmol/L, x ± s)	1.51 ± 0.57	1.55 ± 0.76	t = −0.482	0.630
CK-MB [ng/mL, M (P25, P75)]	1.50 (1.00, 2.50)	2.70 (1.30, 5.70)	Z = 5.012	<0.001
cTnI [ng/mL, M (P25, P75)]	0.01 (0.00, 0.12)	1.60 (0.13, 5.53)	Z = 10.424	<0.001
Glucose (mmol/L, x ± s)	9.87 ± 3.74	10.78 ± 5.89	t = −0.119	0.905
hs-CRP [mg/L, M (P25, P75)]	1.27 (0.53, 3.01)	26.38 (19.24, 31.86)	Z = 6.393	<0.001
BNP [ng/L, M (P25, P75)]	55.00 (22.00, 110.50)	200.25 (90.90, 747.18)	Z = 8.478	<0.001
EF value (%, x ± s)	62.00 ± 8.326	56.83 ± 23.54	t = 4.110	<0.001
Type of NSTEACS (n, %)			χ^2^ = 92.498	<0.001
NSTEMI	204 (29.14)	80 (77.67)		
UAP	496 (70.86)	23 (22.33)		
Number of stenosed coronary vessels (*n*, %)			χ^2^ = 7.956	0.019
1-vessel disease	478 (68.29)	56 (54.37)		
2-vessel disease	110 (15.71)	22 (21.36)		
3-vessel disease	112 (16.00)	25 (24.27)		
Multi-vessel CAD (n, %)	222 (31.71)	47 (45.63)	χ^2^ = 9.044	0.003
GRACE score (x ± s)	99.05 ± 21.26	120.08 ± 36.06	t = −8.420	<0.001
TIMI score (x ± s)	2.14 ± 0.92	2.53 ± 1.11	t = −3.938	<0.001
Gensini score (x ± s)	36.45 ± 31.31	56.00 ± 38.55	t = −4.292	<0.001

Data are expressed as number (percentage), mean ± SD, or median (interquartile range), as specified. PCI, percutaneous coronary intervention; CABG, coronary artery bypass grafting; CPR, cardiopulmonary resuscitation; BP, blood pressure; HR, heart rate; BPM, beats per minute; WBC, white blood cell; Hb, hemoglobin; SCr, serum creatinine; Lac, lactate; CK-MB, creatine kinase-MB; cTnI, cardiac troponin I; hs-CRP, high-sensitivity C-reactive protein; BNP, B-type natriuretic peptide; EF, ejection fraction; NSTEACS, non–ST-segment elevation acute coronary syndrome; NSTEMI, non–ST-segment elevation myocardial infarction; UAP, unstable angina pectoris; CAD, coronary artery disease; TIMI, Thrombolysis in Myocardial Infarction; GRACE, Global Registry of Acute Coronary Events.

### Stratification based on GRACE scores

**Figure 3:**
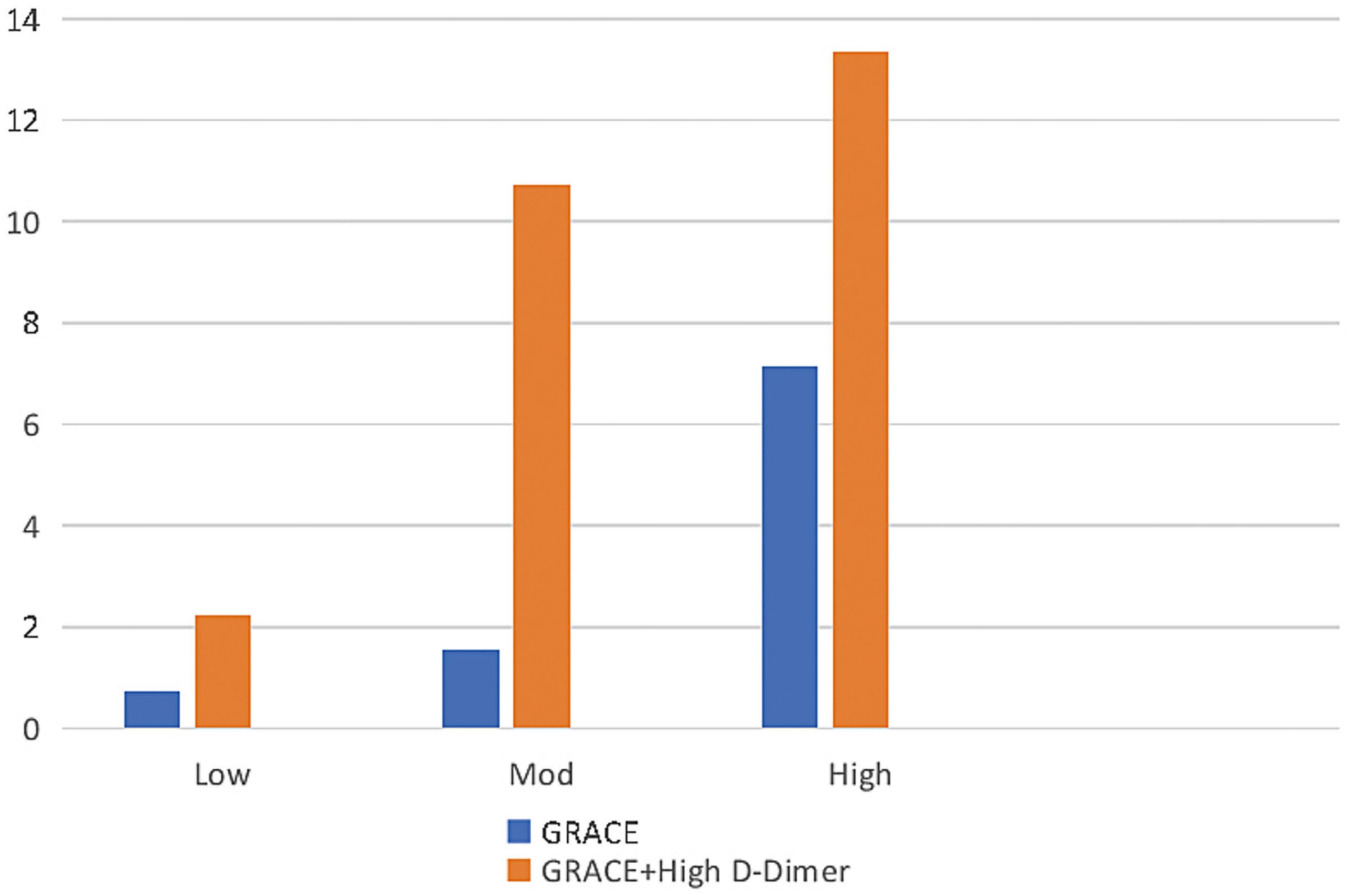
Relationship between d-dimer level and in-hospital mortality rate among patients with different GRACE scores. Percentage of deaths among all patients (blue bars) with different risks of in-hospital death based on GRACE scores (Low, low risk [*n* = 553]; Mod, moderate risk [*n* = 194]; High, high risk [*n* = 56]). Among patients with high d-dimer levels (orange bars), the risk of in-hospital mortality is principally increased in the high- and moderate-risk groups. GRACE, Global Registry of Acute Coronary Events.

Patients were also divided into 3 groups based on their GRACE scores as follows: low risk (≤108 points), moderate risk (109–140 points), and high risk (>140 points). In all 3 groups, the incidence of in-hospital mortality increased with d-dimer elevation (Figure 3). In the low-risk group, the in-hospital mortality rate increased from 0.72 to 2.22% with high d-dimer levels. In the moderate-risk group, the in-hospital mortality rate increased from 1.55 to 10.71%, while in the high-risk group, the in-hospital mortality rate increased from 7.14 to 13.33% with high d-dimer levels.

### Stratification based on TIMI scores

**Figure 4:**
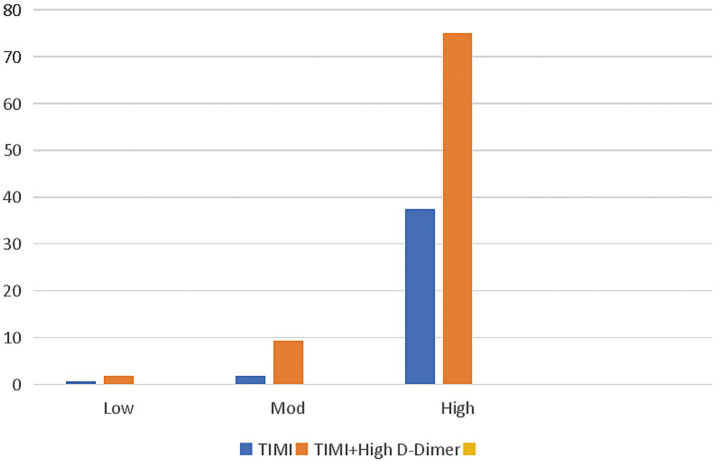
Relationship between d-dimer level and in-hospital mortality rate among patients with different TIMI scores. Percentage of deaths among all patients (blue bars) with different risks of in-hospital death based on TIMI scores (Low, low risk [*n* = 519]; Mod, moderate risk [*n* = 276]; High, high risk [*n* = 8]). Among patients with high d-dimer levels (orange bars), the risk of in-hospital mortality is principally increased in the high- and moderate-risk groups. TIMI, Thrombolysis in Myocardial Infarction.

Next, we divided the patients into 3 groups based on their TIMI scores: low risk (0–2 points), moderate risk (3–4 points), and high risk (5–7 points). In all 3 TIMI groups, patients with elevated d-dimer levels had higher in-hospital mortality rates than patients without elevated d-dimer levels (Figure 4). In the low-risk group, the in-hospital mortality increased from 0.58 to 1.79% with d-dimer elevation, whereas in the moderate- and high-risk groups, the in-hospital mortality increased from 1.81 to 9.30% and 37.50 to 75.00%, respectively, with d-dimer elevation.

### Stratification based on extent of CAD

To evaluate the association between d-dimer levels and the extent of CAD in NSTEACS patients, we categorized patients into 3 groups: 1-vessel disease, 2-vessel disease, and 3-vessel disease groups. We found statistically significant differences (*p* < 0.005) in d-dimer levels and Gensini scores among the 3 groups. Specifically, d-dimer levels were significantly lower in the single-vessel disease group than in the multi-vessel disease group (2-vessel + 3-vessel disease; *p* < 0.005). Furthermore, Gensini scores were significantly lower in the 1-vessel and 2-vessel disease groups than in the 3-vessel disease group (*p* < 0.005; Table 4). However, no significant differences were observed in GRACE and TIMI scores among the 3 groups.

**Table 4:** Comparison of d-dimer level, GRACE score, TIMI score, and Gensini score among patients with different numbers of stenosed coronary vessels.

Parameter	1-vessel disease	2-vessel disease	3-vessel disease	F/Z value	*p* values			
					(1- vs. 2- vs. 3-vessel disease)	(1- vs. 2-vessel disease)	(1- vs. 3-vessel disease)	(2- vs. 3-vessel disease)
D-dimer	31.46 (12.62, 86.91)	70.39 (27.09, 145.45)	71.64 (21.07, 194.41)	30.433	<0.001	<0.001	<0.001	0.743
Gensini score	18.34 ± 16.75	40.65 ± 25.15	69.08 ± 31.59	170.119	<0.001	<0.001	<0.001	<0.001
TIMI score	2.19 ± 0.97	2.14 ± 0.92	2.26 ± 0.93	0.558	0.573	0.655	0.397	0.307
GRACE score	101.01 ± 24.95	102.23 ± 22.97	104.13 ± 25.21	0.902	0.406	0.610	0.187	0.529

TIMI, Thrombolysis in Myocardial Infarction; GRACE, Global Registry of Acute Coronary Events.

### Factors associated with multi-vessel CAD

Univariable logistic regression analysis showed that high Gensini scores [OR: 1.077, *p* < 0.001, 95% CI: 1.061–1.093) and high d-dimer levels [OR: 1.016, *p* = 0.007, 95% CI: 1.010–1.022) were associated with the presence of multi-vessel CAD (2-vessel + 3-vessel disease; Table 5). GRACE score and TIMI score did not enter the regression equation.

**Table 5:** Univariable logistic regression analysis of d-dimer level, Gensini score, TIMI score, and GRACE score for the occurrence of multi-vessel (2-vessel + 3-vessel) coronary artery disease.

Variable	β	SE	Wald value	*P*-value	OR	95% CI
Gensini score	0.074	0.008	93.951	<0.001	1.077	(1.061, 1.093)
D-dimer	0.016	0.003	7.322	0.007	1.016	(1.010, 1.022)

GRACE score and TIMI score did not enter the regression equation. SE, standard error; OR, odds ratio; 95% CI, 95% confidence interval; TIMI, Thrombolysis in Myocardial Infarction; GRACE, Global Registry of Acute Coronary Events.

### Correlation analysis

**Figure 5:**
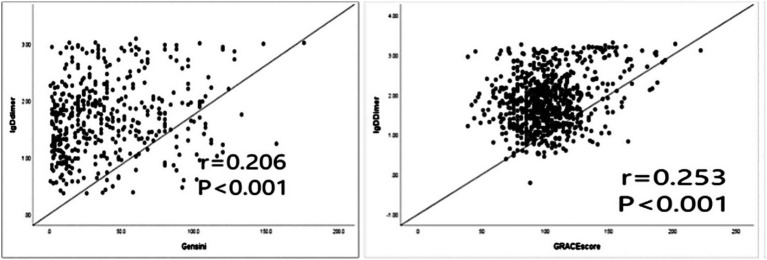
Correlation analysis of lgd-dimer values with Gensini score and GRACE score. LgD-dimer, log-transformed d-dimer values; GRACE, Global Registry of Acute Coronary Events.

To achieve a normal distribution, the d-dimer values were transformed to logarithmic values (lgd-dimer). The lgd-dimer values were positively correlated with Gensini score (r = 0.206, *p* < 0.001), GRACE score (r = 0.253, *p* < 0.001), and TIMI score (r = 0.105, *p* = 0.003). Scatter plots were generated to assess the correlation of lgd-dimer values with GRACE scores and Gensini scores (Figure 5). The correlation with TIMI score was relatively weak, so a scatter plot was not generated for this relationship.

### Comparison of clinical data of patients with different type of NSTEACS

Compared to patients with UAP, patients with NSTEMI had higher in-hospital mortality, higher prevalence of prior percutaneous coronary intervention, faster heart rate, higher white blood cell count, higher blood creatinine levels, higher myocardial marker levels, higher d-dimer levels, higher hs-CRP levels, higher BNP levels, lower EF values, lower blood pressure, higher Gensini scores, higher GRACE scores, higher TIMI scores, and higher rates of multi-vessel CAD (all *p* < 0.005; Supplementary Table S1).

## Discussion

This study revealed that multi-vessel CAD, high d-dimer levels, high TIMI score, and high GRACE scores were independently associated with in-hospital death among patients with NSTEACS. In addition, high d-dimer levels and high Gensini scores were independently associated with multi-vessel CAD. Therefore, our results suggest that d-dimer levels are associated with in-hospital death and the presence of multi-vessel CAD in NSTEACS patients, and may inform clinical decision-making and prognosis assessment.

NSTEACS patients have diverse clinical presentations and significant differences in prognosis. Unstable plaques rupture and form thrombi in the coronary arteries, ultimately leading to the occurrence of ACS (17), and d-dimer is a degradation product of fibrin in the blood, which is associated with thromboembolism. Elevated serum d-dimer levels directly reflect a hypercoagulable and thrombotic state in the body (18), indicating the activation of coagulation and fibrinolysis processes. Thus, d-dimer, as an indicator of coagulation status, may be a useful biomarker for predicting cardiovascular ischemic events (13). Studies have shown that elevated d-dimer levels are a risk factor for coronary heart disease in the general population (13). Multiple studies (17–19) have shown that d-dimer may have good diagnostic and prognostic predictive value for acute myocardial infarction and unstable angina. Zhou et al. (12) have confirmed that high d-dimer levels are associated with adverse outcomes in ACS, and preoperative high serum d-dimer levels are positively correlated with increased mortality in STEMI patients after percutaneous coronary intervention. Two studies on patients with stable CAD (10, 11) have shown that d-dimer levels can predict long-term mortality. A retrospective study including 3,972 ACS patients (20) also indicated that d-dimer is an independent predictor of adverse prognosis in ACS patients undergoing percutaneous coronary intervention. When d-dimer levels are combined with GRACE scores or TIMI scores, their ability to predict the risk of patient mortality is significantly improved (10, 11). The conclusions drawn in this study are consistent with previous research. Although some studies (10, 11, 20) have investigated the prognostic relevance of d-dimer in patients with ACS or stable angina pectoris, research focusing specifically on NSTEACS patients is limited. Furthermore, there is relatively scant research on the correlation between d-dimer levels and the severity of CAD (21, 22), with inconsistent results. The present study is the first to examine the correlation of d-dimer levels with in-hospital mortality as well as the extent of CAD in a cohort of NSTEACS patients. The study found that d-dimer is independently associated with in-hospital mortality in patients with NSTEACS, and for every 1-standard deviation increase in the d-dimer level, the risk of in-hospital death increased by 1.003 times (OR: 1.003, *p* = 0.033, 95% CI: 1.000–1.006). GRACE scores and TIMI scores are the most commonly used scores for predicting prognosis in NSTEACS patients; despite their limitations, they continue to be widely used and are recommended by various guidelines (3, 23, 24). This study found that the area under the ROC curve for d-dimer levels predicting in-hospital death was 0.781 (*p* = 0.001, 95% CI: 0.634, 0.927), which was between the AUCs of GRACE scores (0.672, *p* = 0.050, 95% CI: 0.459, 0.885) and TIMI scores (0.785, *p* = 0.001, 95% CI: 0.649, 0.920). D-dimer levels were positively correlated with other independent risk factors affecting in-hospital death; for instance, lgd-dimer values were correlated with Gensini scores (r = 0.206, *p* < 0.001), GRACE scores (r = 0.253, *p* < 0.001), and TIMI score (r = 0.105, *p* = 0.003). This further demonstrates that d-dimer levels can be used as a potential factor influencing in-hospital mortality. We also found that patients classified into the low-risk group by GRACE and TIMI scores demonstrated increased in-hospital mortality when they had elevated d-dimer levels. Similarly, moderate-risk patients with elevated d-dimer levels showed significantly increased in-hospital mortality. Particularly for patients with a moderate risk according to the GRACE scores, elevated d-dimer levels were associated with an in-hospital mortality rate approaching that of high-risk patients. Therefore, moderate-risk patients according to GRACE scores who have elevated d-dimer levels should receive treatments equivalent to those used for high-risk patients.

In the chronic phase of atherosclerosis, changes in hemostasis and disturbances in fibrinolysis can be detected. Thrombus formation in atherosclerotic lesions, particularly in ruptured plaques, plays a central role in “atherothrombosis” (25). Approximately half of all patients with acute myocardial infarction exhibit multi-vessel CAD, which is associated with higher short-term and long-term mortality rates, and higher incidence rates of acute myocardial infarction than single-vessel CAD (26, 27). In patients with acute myocardial infarction complicated by cardiogenic shock, the rate of triple-vessel CAD is even higher. These patients present with more complex coronary artery lesions, which make successful surgical revascularization challenging, leading to prolonged procedure time and increased complications, and ultimately resulting in higher mortality. Anticipating the possibility of complex CAD and taking preventive measures such as the preoperative implantation of mechanical assist devices, may reduce the occurrence of deaths to some extent. Currently, few indicators have been identified for predicting the number of vessels affected by CAD. In clinical practice, the severity of CAD is often evaluated using the Gensini score, which considers the coronary anatomy, arterial morphology, and severity of stenosis (28). Severe CAD, as indicated by a Gensini score ≥ 20, is associated with short- and long-term cardiovascular risk (29, 30). A small sample study (21) (312 patients with CAD) found that d-dimer levels were not associated with the severity of CAD, as assessed by the number of affected coronary arteries, but rather with the presence of CAD in patients with stable angina pectoris. Conversely, another study (22) involving 2,209 patients with coronary heart disease found that the tertile with elevated d-dimer level was significantly associated with increased coronary stenosis, as assessed using Gensini scores. Additionally, the plasma d-dimer levels increased with the severity of CAD, as evaluated by the number of diseased coronary arteries. Due to the conflicting findings reported in the literature, further discussion and investigation are necessary to clarify the association between elevated d-dimer level and multi-vessel CAD as assessed using Gensini scores. In this study, a positive correlation was found between d-dimer levels and Gensini scores (r = 0.206, *p* < 0.001). Patients in the high d-dimer group had a significantly higher proportion of multi-vessel disease and higher Gensini scores than those in the low d-dimer group (*p* < 0.05). Both d-dimer levels and Gensini scores were lower in the single-vessel disease group than in the multi-vessel disease group (*p* < 0.05). Univariable analysis revealed that the risk of multi-vessel CAD increased by 1.016 times for every 1-standard deviation increase in the d-dimer level (*p* = 0.007, 95% CI: 1.010–1.022). Similarly, for every 1-point increase in the Gensini score, the risk of multi-vessel CAD increased by 1.077 times (*p* < 0.001, 95% CI: 1.061–1.093). This finding further supports the association between d-dimer levels and multi-vessel CAD.

Studies have shown that elevated d-dimer levels in ACS patients are associated with an increased risk of complications during hospitalization as well as in the short- and long-term (22, 31). In addition, d-dimer has been associated with no-reflow phenomena (8, 9, 19), suggesting impaired myocardial perfusion despite successful coronary artery opening. In contrast to these earlier findings, our study specifically examines the association between d-dimer levels and in-hospital death among patients with NSTEACS, which, as a subset of ACS, has not been studied extensively enough in this context. While previous studies (8, 9, 19, 22, 31) have focused on a broader range of complications and no-reflow phenomena, our study provides a practical tool for risk stratification in clinical practice by identifying a clear cut-off value (504 ng/mL) using the Youden index. In addition, our study highlights the independent association between elevated d-dimer levels and the extent of multi-vessel CAD, which was not explored in depth in the earlier literature. Our study deepens our understanding of the role of d-dimer in patients with NSTEACS and provides a more tailored approach to identifying high-risk individuals.

In summary, as a single blood test parameter, d-dimer can provide rapid results at the time of patient admission, potentially offering more immediate risk stratification in scenarios requiring quick decision-making, such as the emergency department. D-dimer directly reflects the degree of hypercoagulability and secondary hyperfibrinolysis in the body, providing direct biological evidence for the core pathological process of thrombosis in NSTEACS. Our findings suggest that this mechanism-specific indicator complements conventional risk scores and may contribute to a more comprehensive risk assessment. We plan to conduct further related randomized controlled trials to evaluate the clinical utility of d-dimer levels in the risk stratification of NSTEACS patients.

This study aimed to preliminarily assess the association of d-dimer levels with in-hospital death and extent of CAD among NSTEACS patients. However, it must be emphasized that the findings of this study should be considered exploratory and hypothesis-generating. Due to the extremely low incidence of in-hospital mortality (1.37%), the statistical power was limited. To mitigate the risk of overfitting associated with a low event-per-variable ratio in conventional multivariable modeling, we employed univariable logistic regression, focusing on a select few clinically relevant variables, including d-dimer, GRACE score, TIMI score, and Gensini score. Consequently, while we observed some significant associations, we explicitly avoid claiming that d-dimer levels are a robust predictor of in-hospital death or multi-vessel CAD among patients with NSTEACS. Instead, our goal is to report these preliminary associations to provide clues for future research. The potential relationships identified here lay the necessary groundwork for subsequent studies; however, they must undergo external validation through larger, prospectively designed studies employing advanced statistical methods such as penalized regression before any clinical application can be considered.

## Conclusion

This study found that the higher the d-dimer level in NSTEACS patients, the higher the risk of in-hospital death and the higher the probability of multi-vessel CAD. Our findings suggest that d-dimer levels may be associated with the risk of in-hospital death and multi-vessel CAD in NSTEACS patients. However, given the limited number of events, further research is needed to validate these findings.

## Limitations

This study has several important limitations that should be considered when interpreting its findings. First, the retrospective, single-center design inherently introduces potential for selection bias. The lack of a systematic comparison with the 200 excluded patients (who did not undergo angiography) prevents a formal assessment of the selection bias introduced, but our study population likely represents a select group with a higher clinical suspicion of CAD and fewer comorbidities. This, combined with the predominance of middle-aged men (72%), limits the generalizability of our results to the broader NSTEACS population. Second, concerning statistical power, the low endpoint event rate resulted in a low event-per-variable ratio, increasing the risk of model overfitting. While we present conventional univariable analyses as a preliminary exploration, we acknowledge that penalized regression methods would be more appropriate and that the use of composite endpoints should be explored in future studies. Third, several key analytical constraints could not be addressed: (1) We could not account for indication bias, and treatment differences confounded by d-dimer levels may have influenced the outcomes. (2) The use of a uniform d-dimer threshold, without age adjustment, is a recognized limitation for risk stratification. (3) Important clinical confounders such as renal function, Killip class, anticoagulant therapy, and inflammatory markers were not included in the analysis. Furthermore, the small number of cases precluded meaningful sensitivity analyses for infections, inflammatory comorbidities, or early deaths, and we were unable to stratify by treatment strategy or incorporate other prognostic biomarkers. Consequently, our findings are hypothesis-generating and must be validated in large, prospective, multi-center studies designed to address these specific limitations.

## Data Availability

The original contributions presented in the study are included in the article/Supplementary material, further inquiries can be directed to the corresponding author.
