# Supplementary material for: Association of d-dimer levels with in-hospital death and multi-vessel coronary artery disease in patients with non–ST-segment elevation acute coronary syndrome
Source: Front Med (Lausanne). 2025 Nov 19;12:1680631. doi: 10.3389/fmed.2025.1680631 (PMC12673484; doi:10.3389/fmed.2025.1680631)
Supplement: Supplementary file 1 [file Table_1.pdf]

Supplementary Table S1 Demographic and clinical characteristics and laboratory results of patients with different type of NSTEACS

**Parameter**	**NSTEMI (n = 284)**	**UAP (n = 519)**	**t/χ^2/Z**	***P***
In-hospital death (n, %)	10 (3.5)	1 (0.19)	χ^2 =15.051	<0.001
Age (years, x ± s)	60.52 ± 11.51	59.71 ± 8.94	t = -1.118	0.264
Male sex (n, %)	210 (73.94)	370 (71.29)	χ^2 = 0.644	0.422
Smoking (n, %)	124 (43.66)	207 (39.88)	χ^2 = 1.838	0.399
Hypertension (n, %)	169 (59.51)	322 (62.04)	χ^2 = 0.497	0.481
Diabetes (n, %)	91 (32.04)	151 (29.09)	χ^2 = 0.758	0.384
Hyperlipidemia (n, %)	60 (21.13)	149 (28.71)	χ^2 = 4.488	0.034
Cerebrovascular disease (n, %)	32 (11.27)	53 (10.21)	χ^2 = 0.200	0.655
Family history of early-onset coronary heart disease (n, %)	1 (0.35)	2 (0.39)	χ^2 = 0.006	0.939
Previous PCI (n, %)	27 (23.57)	148 (19.42)	χ^2 = 39.411	<0.001
Previous CABG (n, %)	7 (2.40)	7 (1.35)	χ^2 = 1.324	0.250
Pre-hospital CPR (n, %)	0 (0.00)	1 (0.19)	χ^2 = 0.006	0.939
BP (mm Hg, x ± s)	127.11 ± 19.55	129.86 ± 17.50	t = -2.043	0.041
HR (BPM, x ± s)	73.55 ± 16.42	68.80 ± 9.91	t = 4.034	<0.001
WBC ($\times 10^9$/L, x ± s)	7.62 ±2.55	6.62 ± 1.68	t = 6.619	<0.001
Hb (g/L, x ± s)	136.18 ± 20.59	140.51 ± 17.31	t = -3.157	0.002
SCr (μmol/L, x ± s)	91.34 ± 53.84	80.40 ± 58.38	t = 2.608	0.009
Lac (mmol/L, x ± s)	1.58 ± 0.66	1.49 ± 0.56	t = 1.541	0.124
CK-MB [ng/mL, M (P25, P75)]	3.20 (1.50, 8.70)	1.30 (1.00, 1.90)	Z = 12.389	<0.001
cTnI [ng/mL, M (P25, P75)]	0.94 (0.14, 2.77)	0.00 (0.00, 0.001)	Z = 19.628	<0.001
Glucose [mmol/L, M (P25, P75)]	6.04 (5.33, 8.24)	6.06 (5.20, 7.42)	Z = 1.233	0.217
hs-CRP [mg/L, M (P25, P75)]	3.95 (1.40, 15.17)	1.14 (0.50, 2.86)	Z = 10.402	<0.001
BNP [ng/L, M (P25, P75)]	124.00 (51.00, 298.00)	39.00 (18.00, 81.50)	Z = 10.835	<0.001
D-dimer [ng/mL, M (P25, P75)]	148.77 (50.90, 588.10)	41.65 (17.51, 102.41)	Z =10.796	0.001
EF value (%, x ± s)	58.88 ± 16.14	62.67 ± 7.75	t = - 4.312	<0.001
Number of stenosed coronary vessels (n, %)			χ^2 = 24.810	<0.001
1-vessel disease	158 (55.63)	376 (72.45)		
2-vessel disease	57 (20.07)	75 (14.45)		
3-vessel disease	69 (24.30)	68 (13.10)		
Multi-vessel CAD (n, %)	126 (44.44)	143 (27.55)	χ^2 = 23.292	<0.001
GRACE score (x ± s)	111.50 ± 31.59	96.40 ± 17.80	t = 8.664	<0.001
TIMI score (x ± s)	2.37 ± 1.02	2.10 ± 0.904	t = 3.869	<0.001
Gensini score (x ± s)	49.19 ± 32.60	34.32 ± 32.42	t = -3.757	<0.001

Data are expressed as number (percentage), mean ± SD, or median (interquartile range), as specified.

PCI, percutaneous coronary intervention; CABG, coronary artery bypass grafting; CPR, cardiopulmonary resuscitation; BP, blood pressure; HR, heart rate; BPM, beats per minute; WBC, white blood cell; Hb, hemoglobin; SCr, serum creatinine; Lac, lactate; CK-MB, creatine kinase-MB; cTnI, cardiac troponin I; hs-CRP, high-sensitivity C-reactive protein; BNP, B-type natriuretic peptide; EF, ejection fraction; NSTEACS, non–ST-segment elevation acute coronary syndrome; NSTEMI, non–ST-segment elevation myocardial infarction; UAP, unstable angina pectoris; CAD, coronary artery disease; TIMI, Thrombolysis in Myocardial Infarction; GRACE, Global Registry of Acute Coronary Events
